# Anti-inflammatory potential of *aspergillus unguis* SP51-EGY: TLR4-dependent effects & chemical diversity via Q-TOF LC-HRMS

**DOI:** 10.1186/s12896-024-00890-1

**Published:** 2024-09-18

**Authors:** Soad Nasr, Abdelhameed S. Dawood, Amal Mosad Ibrahim, Mohamed S. Abdel-Aziz, Walid Fayad, Anwar Abdelnaser, Faten K. Abd EL-Hady

**Affiliations:** 1 Institute of Global Health and Human Ecology, School of Sciences and Engineering, The American University in Cairo (AUC), P.O. Box: 74, Cairo, 11835 Egypt; 2 Biochemical Engineering Department, Faculty of Energy and Environmental Engineering, The British University in Egypt, Suez Desert Road, P.O. Box: 43, El-Shorouk City, Cairo, 11837 Egypt; 3 Chemistry of Natural and Microbial Products Department, National Research Centre, Giza, 12622 Egypt; 4 Department of Microbial Chemistry, National Research Centre, Giza, 12622 Egypt; 5 Drug Bioassay-Cell Culture Laboratory, Pharmacognosy Department, National Research Centre, Giza, 12622 Egypt

**Keywords:** *Aspergillus unguis* SP51-EGY, TLR4, Anti-inflammatory, Macrophages, Q-TOF LC-HRMS

## Abstract

Inflammation serves as an intricate defense mechanism for tissue repair. However, overactivation of TLR4-mediated inflammation by lipopolysaccharide (LPS) can lead to detrimental outcomes such as sepsis, acute lung injury, and chronic inflammation, often associated with cancer and autoimmune diseases. This study delves into the anti-inflammatory properties of “*Aspergillus unguis* isolate SP51-EGY” on LPS-stimulated RAW 264.7 macrophages. Through real-time qPCR, we assessed the expression levels of pivotal inflammatory genes, including iNOS, COX-2, TNF-α, and IL-6. Remarkably, our fungal extracts significantly diminished NO production and showed noteworthy reductions in the mRNA expression levels of the aforementioned genes. Furthermore, while Nrf2 is typically associated with modulating inflammatory responses, our findings indicate that the anti-inflammatory effects of our extracts are not Nrf2-dependent. Moreover, the chemical diversity of the potent extract (B Sh F) was elucidated using Q-TOF LC-HRMS, identifying 54 compounds, some of which played vital roles in suppressing inflammation. Most notably, compounds like granisetron, fenofibrate, and umbelliprenin were found to downregulate TNF-α, IL-1β, and IL-6 through the NF-κB signaling pathway. In conclusion, “*Aspergillus unguis* isolate SP51-EGY”, isolated from the Red Sea, Egypt, has been unveiled as a promising TLR4 inhibitor with significant anti-inflammatory potentials, presenting novel insights for their potential therapeutic use in inflammation.

**Supplementary Information:**

The online version contains supplementary material available at 10.1186/s12896-024-00890-1.

## Graphical abstract

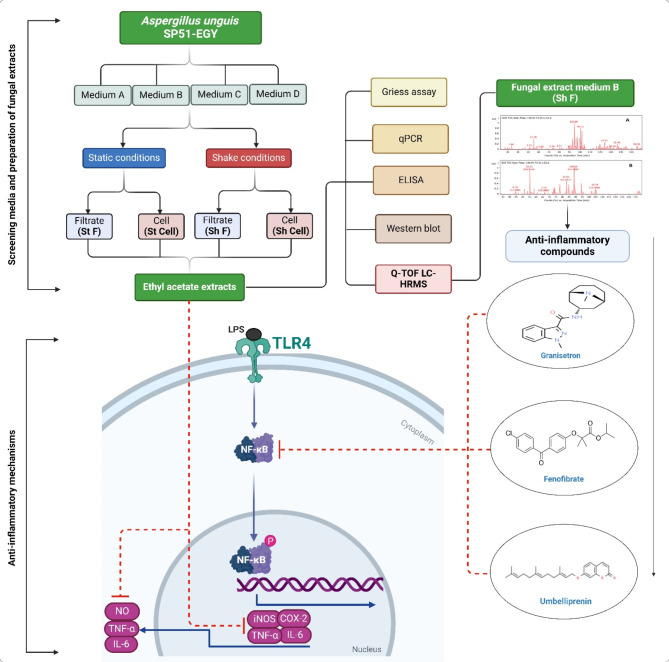

## Introduction

After injury or infection, an innate immune response arises in the body, leading to inflammation. Inflammation is considered a complex defense machinery to repair tissues in the body [1]. The first line of defense to eradicate infections is macrophages. Toll-like receptors (TLRs) signaling pathways are activated by macrophages via lipopolysaccharide (LPS) with TLR4 ligates. This serves as an activating signal which leads to the initiation of intracellular pathways [2]. The first inflammatory response involves the release of prostaglandins, histamine, and nitric oxide (NO) by inducible nitric oxide synthase (iNOS), which results in vasodilation, increased blood flow, and recruitment of leukocytes. Following activation of immune cells, pro-inflammatory cytokines like interleukin-6 (IL-6), interleukin-1 (IL-1), and tumor necrosis factor-alpha (TNF-α) escalate leukocyte permeability in the vascular regions by increasing leukocyte adhesion to endothelial cells. Homeostasis is necessary during inflammation as prolonged inflammation could lead to serious health issues [2, 3]. In addition, the nuclear factor erythroid 2-related factor 2 (Nrf2) is the primary redox homeostasis regulator. Nrf2 plays a crucial role in protecting cells from inflammation and oxidative stress by regulating the expression of phase II detoxification enzymes and oxidative stress response proteins, such as heme oxygenase-1 (HO-1), and oxidative stress-induced growth inhibitor 1 (OSGIN1) [4, 5].

Natural products have been used since the beginning of time for healing applications. Currently, natural products are being used for the treatment of inflammatory diseases. Previously discovered herbal extracts have been shown to have key effects on TLR4 signaling pathways, a significant pathway for a pro-inflammatory response in the body [6]. In addition, natural extracts have been shown to employ their anti-inflammatory effects by regulating the expression of pro-inflammatory cytokines in LPS-induced inflammatory response [7].

Marine-associated fungi have an excellent provenance for secondary metabolites, many of which have diverse biological activities and highly complicated structures, making them hard to provide economically via chemical synthesis [8]. Furthermore, marine microorganisms can be cultured easily, which spreads high reproducibility and is a continual source of natural products [9]. The *Aspergillus* genus contains more than 300 species that live in a variety of habitats, of which the marine-derived species produce variable structures of secondary metabolites, like alkaloids, phenolics, terpenoids, and peptides with prominent biological effects such as cytotoxic, antimicrobial, and anti-inflammatory activities [10]. However, little is known about the chemical constituents and biological activities of “*Aspergillus unguis* isolate SP51-EGY” from the Red Sea, except that our previous work reported its antidiabetic effect [11]. Liquid chromatography-high-resolution mass spectrometry (LC-HRMS) is the dominant way to attain pure natural products for structure elucidation and evolution into therapeutic agents. In recent years, LC-HRMS use in natural product chemistry has grown in popularity, allowing for various analytical platforms for non-targeted, targeted, and suspect screening. LC-HRMS provides valuable structural input for detecting and exploring chemical substances identified from natural products [12].

This study aimed to find the highly effective anti-inflammatory fungal extract and to identify its secondary metabolites using LC-HRMS, which plays an essential role in determining accurate masses and is used for comprehensive analysis in both positive and negative ionization modes to identify chemical compositions. This study proved that LC-HRMS is an efficacious and powerful analytical appliance for characterizing most of the identified compounds of “*Aspergillus unguis* isolate SP51-EGY” from Red Sea, Egypt.

## Materials and methods

### Materials

The RAW 264.7 cell line was purchased from the American Type Culture Collection (ATCC TIB-71; RRID: CVCL_0493). LPS (Escherichia coli O111:B4) was purchased from Sigma-Aldrich (St. Louis, MO, USA). QIAzol Lysis Reagent and Nuclease-free water were obtained from Qiagen (Hilden, Germany). Dimethyl Sulfoxide (DMSO), Methanol, Chloroform, Isopropanol, and Ethanol were of HPLC grade and were all purchased from SERVA (Heidelberg, Germany). Cyclooxygenase-2 (COX-2) Polyclonal Antibody, Mouse TNF-α, and Mouse IL-6 ELISA Kits were purchased from Elabscience (Wuhan, China). Dulbecco’s Modified Eagle Medium (DMEM), Phosphate Buffered Saline (PBS), Fetal Bovine Serum (FBS), Penicillin-Streptomycin (Pen/Strep), (3-(4, 5-dimethylthiazol-2-yl)-2, 5-diphenyltetrazolium bromide (MTT), Griess Reagent Kit, RevertAid First Strand cDNA Synthesis Kit, PowerUp™ SYBR™ Green Master Mix, mRNA primers (iNOS, COX-2, TNF-α, IL-6, COX-2 and GAPDH), Pierce™ BCA Protein Assay Kit, Pierce™ 20X TBS Tween™ 20 Buffer, Blocker™ BSA (10X) in PBS, GAPDH Polyclonal Antibody, Goat Anti-Rabbit IgG (H + L) Secondary Antibody, Horseradish Peroxidase (HRP)-conjugated, and Pierce™ ECL Western Blotting Substrate were all purchased from Thermo Fisher Scientific (Waltham, MA, USA). Cell Lysis Buffer (10X), Protease Inhibitor Cocktail (100X), and Prestained Protein Marker, Broad Range (11–190 kDa) were purchased from Cell Signaling (Danvers, MA, USA). Heme Oxygenase-1 (HO-1), Oxidative Stress-Induced Growth Inhibitor-1 (OSGIN1) mRNA primers were ordered from Synbio Technologies (Monmouth Junction, NJ, USA).

### Sponge materials and molecular identification of the fungal isolate

The Sponge (*Agelas* sp.) was gathered from the coast of Hurghada, Red Sea, Egypt (Shaab al-Ariq latitude, N 27° 25ˊ 08.9˝, E 33° 51ˊ 0.5). Sponge sample was used according to Aboutabl et al. [11]. The fungus was identified as “*Aspergillus unguis* isolate SP51-EGY”. The sequence has been deposited in GenBank with the name *Aspergillus unguis* isolate SP51-EGY and accession number KM203831.1 [11].

### Screening media and preparation of fungal extracts

For the fungus cultivation, four different broth media (A, B, C, and D) were used. The media were made up of the following components (g/L): Medium A (Sabouraud broth) contains dextrose (20 g/L) and peptone (10 g/L); Medium B (Nutrient broth) contains peptone (5 g/L), beef extract (1 g/L), yeast extract (2 g/L), and sodium chloride (6 g/L); Medium C (Potato dextrose broth) contains dextrose (20 g/L) and potato-infusion (200 g/L); Medium D (Malt extract broth) contains yeast extract (3 g/L) and malt extract (17 g/L). Secondary metabolite ethyl acetate extracts from static and shake conditions were abbreviated as: **Sh F** (shake filtrate) extract, **Sh Cell** (shake mycelia) extract, **St F** (static filtrate) extract, and **St Cell** (static mycelia) extract.

### Cell culture

RAW 264.7 cells were cultured in a 5% CO_2_ humidified incubator at 37 °C in DMEM supplemented with 10% heat-inactivated FBS and 1% Pen-Strep (100 units/mL penicillin, and 100 µg/mL streptomycin).

### Determination of NO production using Griess method

RAW 264.7 cells were cultured in a 96-well plate for 2 h at a seeding density of 1 × 10^6^ cells/mL. The cells were then stimulated with LPS (10 ng/mL) and co-treated with different fungal extracts at concentration of 10 µg/mL for 24 h. The NO production in the cell culture medium was determined using the Griess method through mixing 150 µL of the culture supernatant from each well with 130 µL of deionized water and 20 µL of Griess reagent and incubating for 30 min in the dark at room temperature as previously described [13]. The absorbance was measured at 548 nm using SPECTROstar^®^ Nano microplate reader (BMG LABTECH, Germany). NO concentration in each sample was eventually calculated using a NaNO_2_ standard curve.

### Determination of cytotoxicity using MTT assay

To confirm that the anti-inflammatory effect of the fungal extracts was not a result of cytotoxicity, MTT colorimetric assay was performed. On the 96-well plate containing cultured RAW 264.7 macrophages from the Griess experiment, MTT solution was added (1 mg/mL) for 2 h at 37^o^C. MTT solution was then discarded and a volume of 100 µL of DMSO was added in order to solubilize the formed formazan crystals. The absorbance was measured at 540 nm using SPECTROstar^®^ Nano microplate reader. Cell viability percentage was then determined relative to the control group.

### Isolation of total RNA

RAW 264.7 cells were seeded at a density of 1 × 10^6^ cells/mL in 6-well plates and incubated overnight. The cells were then stimulated with LPS (10 ng/mL) and co-treated with different fungal extracts at concentration of 10 µg/mL for 6 h. Total RNA was extracted from the cells using QiAzol lysis reagent. An amount of 1 µg of RNA from each sample was used to synthesize the cDNA using RevertAid First Strand cDNA Synthesis Kit according to manufacturer’s protocol. cDNA was stored in -20 ^o^C for further use in mRNA expression analysis.

### Determination of inflammatory mRNA expression levels using quantitative real-time polymerase chain reaction (qPCR)

The qPCR was used for the determination of inflammatory mRNA expression levels. The cDNA was mixed with forward and reverse primers (Table 1), maxima SYBR green mix, and nuclease free water. The inflammatory genes selected for this experiment are: iNOS, COX-2, TNF-α, and IL-6. All mRNA expression levels were normalized to endogenous control (GAPDH) and the 2^−ΔΔCT^ method was used to evaluate the relative fold mRNA expression levels. The qPCR analysis was performed using ABI Prism 7500 system (Applied Biosystems) under the following conditions: initial holding stage at 95 °C for 10 min, followed by 40 PCR cycles of denaturation at 95 °C for 15 s and annealing/extension at 60 °C for 1 min. Primer design generation was done using NCBI Primer-Blast tool (Table 1).

**Determination of mRNA expression levels of Nrf2-driven ARE genes HO-1 and OSGIN1 in LPS-stimulated RAW 264.7 macrophages**.

The qPCR primers for Nrf2-driven ARE Genes HO-1 and OSGIN1 were used to determine whether the anti-inflammatory activity of the fungal extracts was associated with Nrf2 signaling pathway. The qPCR reactions were subjected to the same thermocycling conditions as the previous experiment. All mRNA expression levels were normalized to endogenous control (GAPDH) and the 2^−ΔΔCT^ method was used to evaluate the relative fold mRNA expression levels.

**Table 1:** mRNA sequences used for qPCR

Target mRNA	Primer sequence (5′–3′)		Tm
iNOS	Forward:	GGAACCTACCAGCTCACTCTGG	63
	Reverse:	TGCTGAAACATTTCCTGTGCTGT	60
COX-2	Forward:	CTCACGAAGGAACTCAGCAC	58
	Reverse:	GGATTGGAACAGCAAGGATTTG	58
TNF-α	Forward:	GAACTCCAGGCGGTGCCTAT	63
	Reverse:	TGAGAGGGAGGCCATTTGGG	63
IL-6	Forward:	GATGCTACCAAACTGGATATAATCAG	55
	Reverse:	CTCTGAAGGACTCTGGCTTTG	58
HO-1	Forward:	CACAGATGGCGTCACTTCGTC	60
	Reverse:	GTGAGGACCCACTGGAGGAG	62
OSGIN1	Forward:	CGGTGACATCGCCCACTAC	62
	Reverse:	GCTCGGACTTAGCCCACTC	62
GAPDH	Forward:	CTTTGTCAAGCTCATTTCCTGG	57
	Reverse:	TCTTGCTCAGTGTCCTTGC	58

### Quantification of the pro-inflammatory cytokine protein levels using enzyme-linked immunosorbent assay (ELISA)

Protein expression levels of the pro-inflammatory cytokines, TNF-α and IL-6, were quantified by pre-coated ELISA plates (Elabscience^®^) according to the manufacturer’s protocol. RAW 264.7 cells were seeded in 6-well plates (1 × 10^6^ cells/mL) and incubated overnight. The cells were then stimulated with LPS (10 ng/mL) and co-treated with different fungal extracts at concentration of 10 µg/mL for 24 h. After incubation, the supernatants were collected, centrifuged at 1000× g for 20 min at 4 °C and then transferred to new microcentrifuge tubes to analyze the level of TNF-α and IL-6 proteins secreted into the cell culture medium. The supernatant was diluted in a ratio of 1:100 for TNF-α and 1:20 for IL-6 in buffered sample diluent. The absorbance was measured at 450 nm using SPECTROstar^®^ Nano microplate reader.

### Western blotting

Protein expression level of COX-2 was determined using Western blotting. RAW 264.7 cells were seeded in 6-well plates (1 × 10^6^ cells/mL) and incubated overnight. The cells were then stimulated with LPS (10 ng/mL) and co-treated with different fungal extracts at concentration of 10 µg/mL for 24 h. Total proteins were extracted from the cells using cell lysis buffer containing a protease inhibitor cocktail, and the protein concentration was measured using Pierce™ BCA Protein Assay Kit. Western blotting was performed using a standard protocol. Briefly, proteins (10 µg) were separated by 10% SDS-PAGE and transferred to a PVDF membrane. After blocking with 5% non-fat dry milk in TBST for 1 h at room temperature, the membrane was incubated with primary antibodies; anti-COX-2 antibody (1:1000 dilution), and anti-GAPDH antibody (1:2500 dilution) overnight at 4 °C. After washing three times with TBST, the membrane was incubated with HRP-conjugated secondary antibody (1:15000 dilution) for 1 h at room temperature. Protein bands were visualized with Pierce™ ECL Western Blotting Substrate using ChemiDoc MP Imaging System (Bio-Rad Laboratories, CA, USA).

### Q-TOF LC-HRMS spectroscopy analysis

For chromatographic separation, a 6530 Q-TOF LC/MS (Agilent Technologies) equipped with an autosampler (G7129A), a Quat. Pump (G7104C), and a Column Comp (G7116A) were used at the Faculty of Pharmacy, Fayoum University. The injection volume was set at 5 L. The analytes were separated on an Agilent Technologies Zorbax RP-18 column (dimensions: 150 mm 3 mm, dp = 2.7 m) at a flow rate of 0.3mL/min. ESI was used to obtain mass spectra in (+) and (-) ionization modes with a capillary voltage of 4500 V. The mass spectra were recorded in the 50–3000 m/z range. The gas temperature and drying gas flow were 200 ^O^C and 8 L/min, respectively. The skimmer and fragmentator voltages were set at 65 and 130 V, respectively, and collision energy was 10 V. The nebulization pressure was 58psig. Elution was with Solvent A (Water 0.1%formic acid) and Solvent B (Acetonitrile 0.1%formic a), the flow rate was 0.3mL/min. Gradient elution started with 2% B, reaches 10% B at 15 min and 20% B at 35 min, 50% B at 60 min, 70% B at 80 min and then 100% B at 100 min.

### Statistical analysis

Statistical significance between groups was determined using one-way analysis of variance (ANOVA) followed by Student–Newman–Keuls post hoc test, where *P*-value < 0.05 was considered statistically significant. The data from three independent experiments are presented as the mean ± standard error of the mean (SE). Comparative analysis between experimental groups was performed using SigmaPlot (Version 14.0; Systat Software, Chicago, IL, USA).

## Results and discussion

### Determination of NO production in LPS-stimulated RAW 264.7 macrophages

Macrophages play a key role in the innate immune response [14, 15]. LPS, a major component in the cell wall of gram-negative bacteria can induce the production of inflammatory mediators, and is frequently used to evaluate the anti-inflammatory effects of drugs [16]. The production of inflammatory mediators including NO is increased in LPS-stimulated macrophages. NO is a bioactive signaling molecule which has a key role in modulating the inflammatory response [17]. Therefore, the anti-inflammatory effect of the fungal extracts was evaluated using LPS-stimulated RAW 264.7 macrophages. The NO production in the cell culture medium was determined using the Griess method and according to the measured NaNO_2_ standard curve. It was shown that NO production was significantly increased in the cell culture medium of LPS-stimulated macrophages (19.8 µM) compared to the control (11.2 µM).

**Fig. 1:**
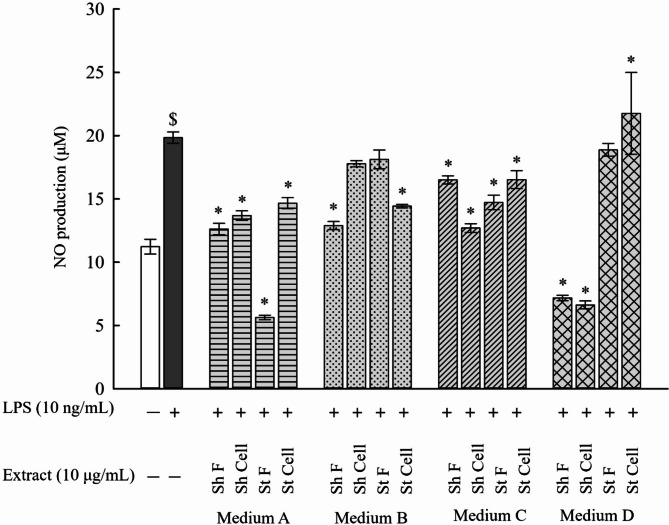
Measurement of NO production of extracts found in medium (A-D) on RAW 264.7 macrophages. After treatment of LPS-stimulated RAW 264.7 macrophages (1 × 10^6^ cells/mL) with 10 µg/mL of extracts in medium (A-D) and cultured overnight. NO production in the cell culture medium was measured using the Griess method. LPS-stimulated cells only showed high significance in comparison to control. All extracts in medium A and medium C showed a significant decrease in NO production in comparison to LPS-stimulated cells. However, Sh Cell and St F extracts in medium B and St F and St Cell in medium D did not show any significant decrease in comparison to LPS-stimulated cells. Results are presented as the mean ± SE (*n* = 3). Statistical significance was calculated by one-way ANOVA followed by Student–Newman–Keuls post-hoc test. $ *P* < 0.05 vs. control cells. * *P* < 0.05 vs. LPS-stimulated cells

As for the extracts in medium (A-D), NO production was significantly reduced in all samples in medium A and C in comparison to LPS-stimulated cells. As for medium B, only Sh F and St Cell extracts significantly reduced NO production, resulting in levels of 12.9 µM and 14.4 µM, respectively, in comparison to LPS-stimulated cells, and for medium D, Sh F and Sh Cell extracts significantly reduced NO production, resulting in levels of 7.2 µM and 6.8 µM, respectively, in comparison to LPS-stimulated cells **(**Fig. 1**)**. These results validate that some of the fungal extracts have significant effect on reducing NO production in LPS-stimulated RAW 264.7 cells and highlight the potential anti-inflammatory effect on RAW 264.7 cells [18].

**Fig. 2:**
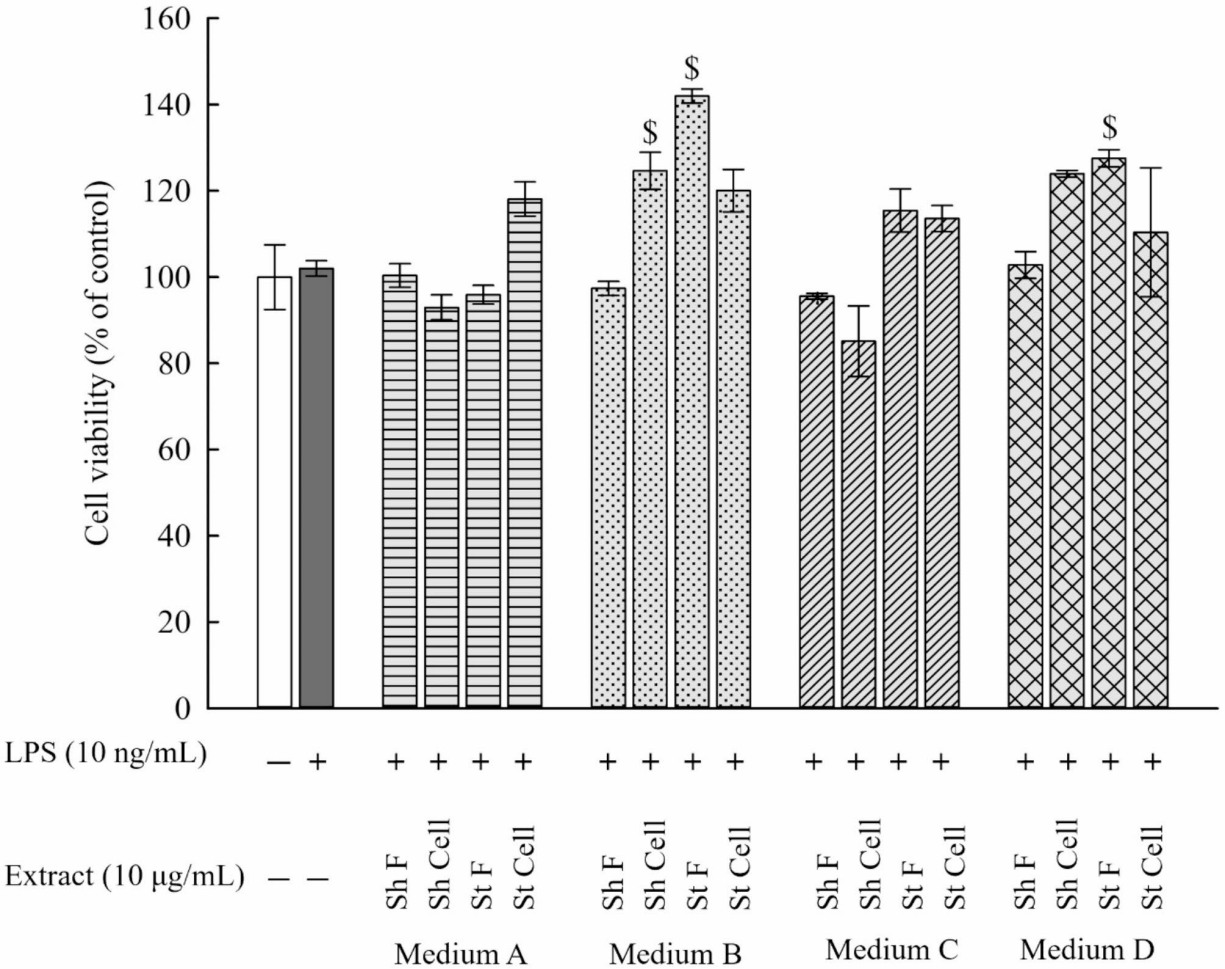
Measurement of cytotoxicity on RAW 264.7 macrophages using MTT assay. After treatment of LPS-stimulated cells with 10 µg/mL of extracts found in medium (A-D) and cultured overnight, cell viability was determined using MTT assay. All extracts in medium (A-D) did not show any cytotoxicity on RAW 264.7 cells. Results are presented as the mean ± SE (*n* = 3). Statistical significance was calculated by one-way ANOVA followed by Student–Newman–Keuls post-hoc test. $ *P* < 0.05 vs. control cells. * *P* < 0.05 vs. LPS-stimulated cells

To ensure that the observed NO reduction was not due to a reduction in cell viability, MTT assay was used to measure the cytotoxicity of RAW 264.7 macrophages after treatment with the extracts (medium A-D). Our results showed that no cytotoxicity was observed at the tested concentrations on the cultured cells. As a result, the reduction in NO production observed in the LPS-stimulated cells after treatment with the extracts was not due to cytotoxicity. The anti-inflammatory effect of these extracts was further experimented on to assess their anti-inflammatory effect using the same concentration (10 µg/mL) of NO production **(**Fig. 2**)**.

### Determination of mRNA expression levels of the pro-inflammatory markers iNOS, COX-2, TNFα, and IL-6 in LPSstimulated RAW 264.7 macrophages

**Fig. 3:**
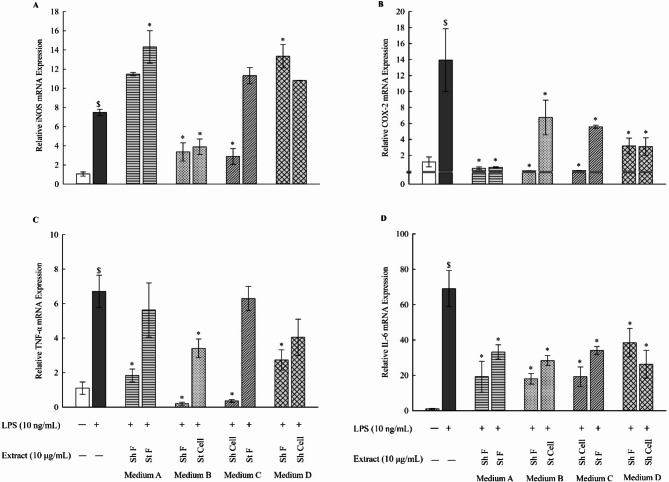
Expression levels of iNOS, COX-2, TNF-α, and IL-6 mRNA in LPS-stimulated RAW 264.7 macrophages. Fungal extracts in medium A-D (10 µg/mL) were used for treatment of LPS-stimulated RAW 264.7 macrophages (LPS concentration: 10 ng/mL). The mRNA levels of iNOS, COX-2, TNF-α and IL-6 were measured using qPCR by the comparative method (2^−ΔΔCT^). Results are presented as the mean ± SE (*n* = 3). Statistical significance was calculated by one-way ANOVA followed by Student–Newman–Keuls post-hoc test. $ *P* < 0.05 vs. control cells. * *P* < 0.05 vs. LPS-stimulated cells

During inflammation, the immune system triggers the production of pro-inflammatory cytokines (TNF-α, IL-6), as well as enzymes (iNOS, COX-2) [19]. Current research is emphasizing the exploration of bioactive compounds with the capacity to suppress the production of these inflammatory mediators. Natural products are gaining attention as potent and safe anti-inflammatory agents, owing to their capability to modulate gene expression of diverse inflammatory mediators, thus offering promising avenues for therapeutic intervention [20]. Hence, to investigate the anti-inflammatory activities of the fungal extracts, the mRNA expression level of iNOS, COX-2, TNF-α, and IL-6 was determined using qPCR (Fig. 3). Overall, eight samples from the different extraction media, with the highest anti-inflammatory action as evidenced by Griess assay, were selected for further anti-inflammatory studies. The results from qPCR further confirmed our previous results obtained from the Griess analysis. For LPS-stimulated macrophages, significant upregulation was observed for iNOS, COX-2, TNF-α, and IL-6 mRNA levels compared to the control. After treatment with the fungal extracts, the mRNA expression levels were significantly reduced. For the iNOS mRNA expression levels, significant reduction was observed for Sh F and St Cell in medium B, and Sh Cell in medium C **(**Fig. 3A**)**. As for COX-2 mRNA expression levels, all samples in medium (A-D) showed a highly significant reduction in COX-2 expression in comparison to LPS-stimulated macrophages **(**Fig. 3B**)**. Furthermore, the following samples showed significant reduction in TNF-α mRNA expression levels: Sh F in medium A, Sh F and St Cell in medium B, Sh Cell in medium C, and Sh F in medium D. However, St F (medium A), St F (medium C) and Sh Cell (medium D) did not show any significant reduction in TNF-α mRNA expression levels **(**Fig. 3C**)**. Finally, for IL-6 mRNA expression levels, all 8 samples in all extraction medium (medium A-D) showed a highly significant reduction in IL-6 mRNA expression levels in comparison to LPS-stimulated macrophages (Fig. 3D**)**. Our findings are in agreement with the results reported by Anh et al. indicating that nitrogen-containing secondary metabolites from *Aspergillus unguis* showed anti-inflammatory activity by suppressing the production of NO and the expression of iNOS and IL-6 in LPS-stimulated RAW 264.7 macrophages [21]. In a previous study conducted by Cao et al., it has also been demonstrated that sterols isolated from *Aspergillus unguis* inhibited the production of inflammatory mediators, including NO and IL-6, along with downregulation of iNOS and IL-6 expressions in LPS-stimulated RAW 264.7 macrophages [22].

### Determination of mRNA expression levels of Nrf2-driven ARE genes HO-1 and OSGIN1 in LPS-stimulated RAW 264.7 macrophages

**Fig. 4:**
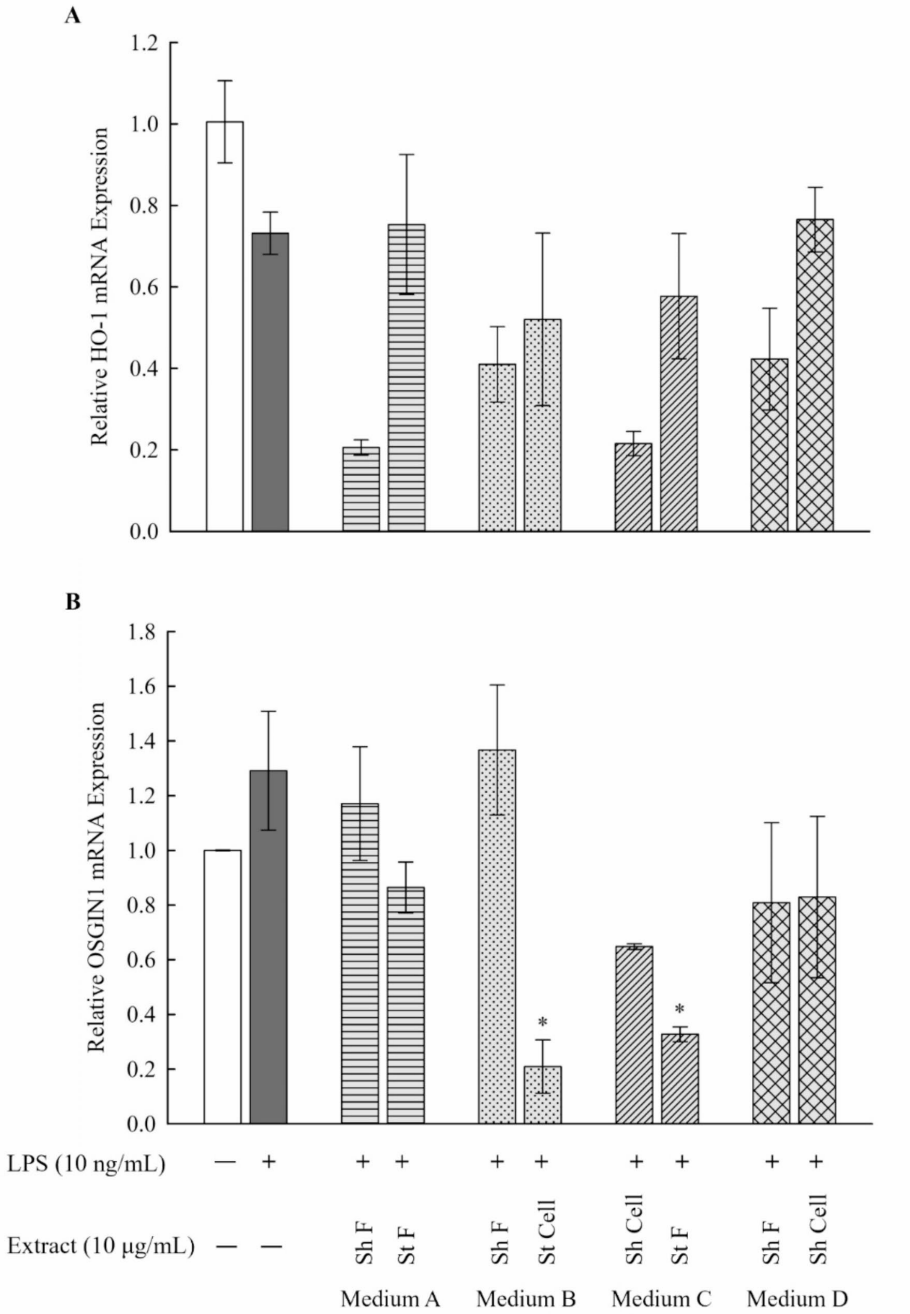
Expression levels of Nrf2-driven ARE genes, HO-1 (**A**) and OSGIN1 (**B**) mRNA in LPS-stimulated RAW 264.7 macrophages. Fungal extracts in medium A-D (10 µg/mL) were used for treatment of LPS-stimulated RAW 264.7 macrophages (LPS concentration: 10 ng/mL). The mRNA levels of HO-1 and OSGIN1 was measured using qPCR by the comparative method (2^−ΔΔCT^). Results are presented as the mean ± SE (*n* = 3). Statistical significance was calculated by one-way ANOVA followed by Student–Newman–Keuls post-hoc test. $ *P* < 0.05 vs. control cells. * *P* < 0.05 vs. LPS-stimulated cells

The Nrf2-antioxidant response element (ARE) signaling pathway, which is recognized as a key regulatory system maintaining the intracellular redox homeostasis, also has a role in reducing inflammation [4]. The Nrf2-ARE activity can be attributed to modulating the expression of antioxidant and detoxifying enzymes, such as HO-1 and OSGIN1 [23, 24]. Additionally, recent studies demonstrated a correlation between the expression of inflammatory mediators, the NF-κB pathway, macrophage metabolism, and the Nrf2/ARE system [25]. To examine whether the inhibitory effect of the selected fungal extracts on LPS-mediated inflammation in RAW 264.7 macrophages was related to activation of the Nrf2-driven genes, the mRNA expression levels of HO-1 and OSGIN1 was analyzed by qPCR. Our data showed no significant upregulation in HO-1 and OSGIN1 mRNA expression between control, LPS-stimulated cells, and treated groups in mediums A-D. This is an indication that both HO-1 and OSGIN1 are not affected by any of the treatments (Fig. 4). These results propose that the anti-inflammatory effects of the fungal extracts are Nrf2-independent.

### Determination of protein levels of the pro-inflammatory cytokines TNFα and IL-6 in LPSstimulated RAW 264.7 macrophages

**Fig. 5:**
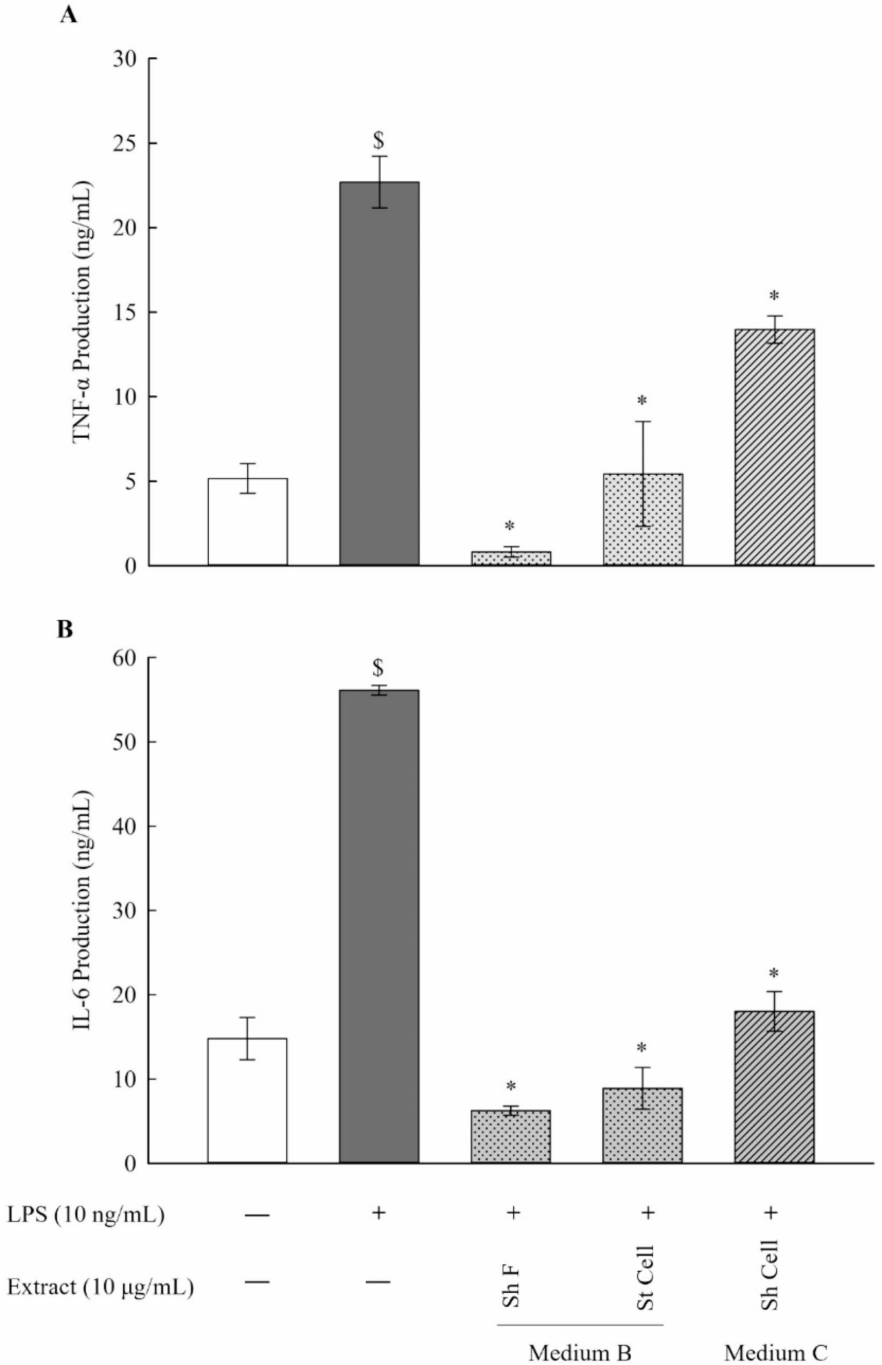
Determination of protein levels of the pro-inflammatory cytokines TNF-α and IL-6 in the cell culture medium of the LPS-stimulated RAW 264.7 macrophages using ELISA. The selected fungal extracts (Sh F and St Cell in medium B and Sh Cell in medium C) showed a significant reduction of the LPS-stimulated upregulation of the pro-inflammatory cytokines TNF-α (**A**) and IL-6 (**B**). Results are presented as the mean ± SE (*n* = 3). Statistical significance was calculated by one-way ANOVA followed by Student–Newman–Keuls post-hoc test. $ *P* < 0.05 vs. control cells. * *P* < 0.05 vs. LPS-stimulated cells

Cytokines are key signaling proteins, which are produced to regulate the interactions between different cell types involved in the immune response [26]. Cytokines are mainly produced by phagocytic cells and natural killer (NK) cells during innate immune responses, while they are mostly secreted by lymphocytes and antigen-presenting cells (APCs) during adaptive immune responses. Therefore, cytokines coordinate the crosstalk between the innate and adaptive immune systems. Among these, TNF-α and IL-6 are the major pro-inflammatory cytokines involved in the inflammatory response [27, 28]. In the present study, the protein expression levels of the pro-inflammatory cytokines TNF-α and IL-6, were assessed by ELISA. This was to confirm that the anti-inflammatory responses observed on the mRNA level carried through to the protein level. A concentration of 10 ng/mL of LPS significantly increased the pro-inflammatory response of RAW 264.7 macrophages as indicated by the elevated protein levels of the assessed cytokines (Fig. 5). However, at concentration of 10 µg/mL of the selected fungal extracts (Sh F and St Cell in medium B and Sh Cell in medium C), significant reduction was observed in protein levels of the pro-inflammatory cytokines TNFα and IL-6 in LPSstimulated RAW 264.7 macrophages.

### Determination of protein expression of COX-2 using western blotting

**Fig. 6:**
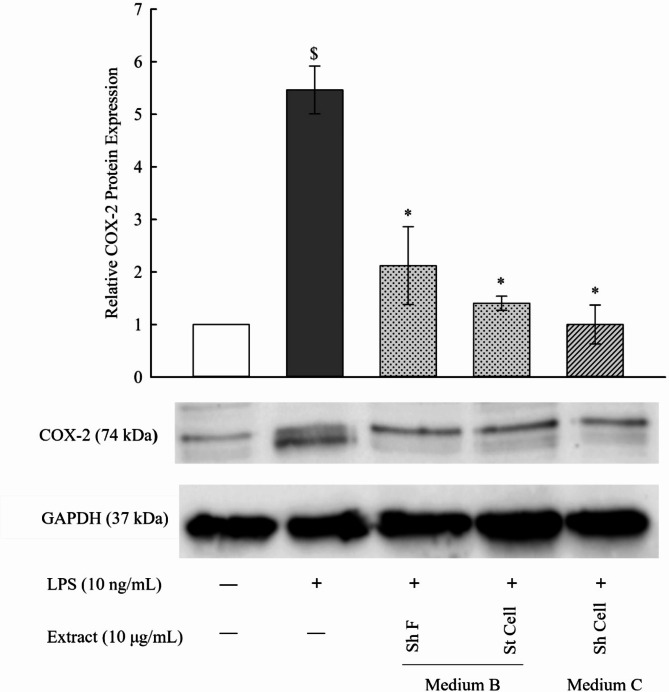
Western immunoblots and derived graphical illustration of the protein expression level for COX-2 in selected fungal extracts (Sh F and St Cell in medium B and Sh Cell in medium C). Results are presented as the mean ± SE (*n* = 2). Statistical significance was calculated by one-way ANOVA followed by Student–Newman–Keuls post-hoc test. $ *P* < 0.05 vs. control cells. * *P* < 0.05 vs. LPS-stimulated cells

The iNOS induction in relation to COX-2 has been formerly reported in previous studies. In this context, it was shown that selective COX-2 inhibitors reduce COX-2-mediated generation of prostacyclin (PGI2). Moreover, PGI2 was demonstrated to induce iNOS expression, resulting in the production of NO. Thus, it is likely that the fungal extracts might have reduced NO production via a selective inhibition of COX-2 [29]. A concentration of 10 ng/mL of LPS significantly increased the pro-inflammatory response of RAW 264.7 macrophages as indicated by the elevated protein levels of COX-2 (Fig. 6). However, at concentration of 10 µg/mL of the selected fungal extracts (Sh F and St Cell in medium B and Sh Cell in medium C), significant reduction was observed in COX-2 protein levels in LPSstimulated RAW 264.7 macrophages.

### Q-TOF LC-HRMS spectroscopy analysis

LC-MS coupled with QTOF is a cutting-edge analytical technique for identifying unknown bioactive compounds in natural product extracts. The key to obtaining pure natural products for structure elucidation and development into therapeutic agents is liquid chromatography-high-resolution mass spectrometry (LC-HRMS).

**Fig. 7:**
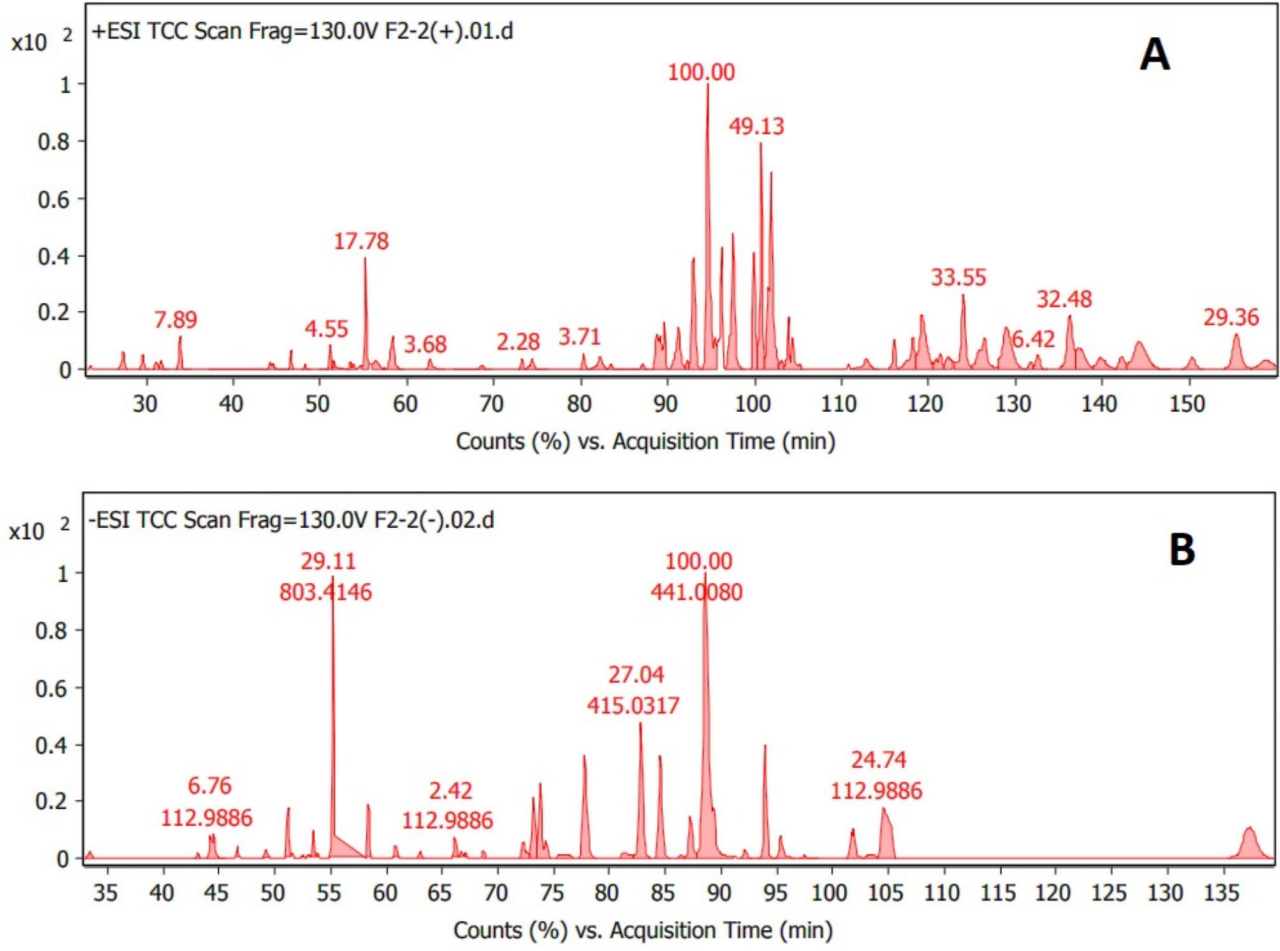
Total compound chromatogram (TCC) of Q-TOF LC-HRMS analysis of (B Sh F extract) for the fungus “*A. unguis* isolate SP51-EGY”; A = in positive, B = in negative ionization mode

**Fig. 8:**
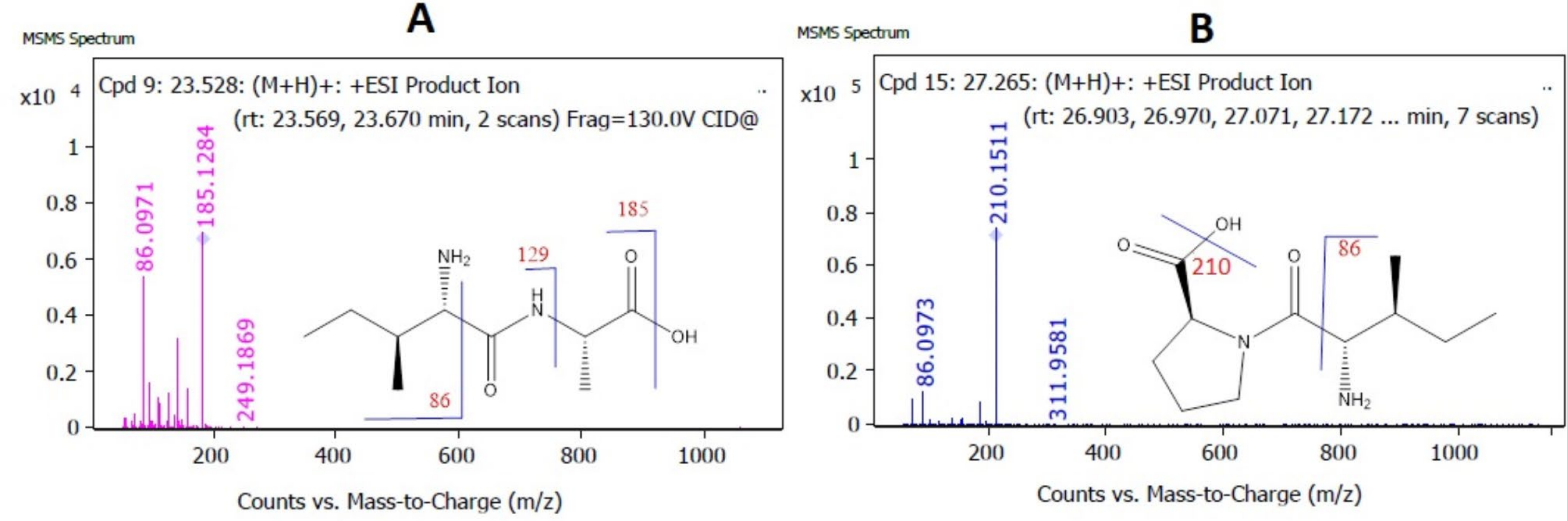
LC-Q-TOF mass spectrum (ESI^**+**^) of peptides and their proposed fragmentation patterns in positive-ion mode. **A** = Isoleucyl-Alanine, [M + H]^**+**^with *m/z***202**) shows product ions with *m/z***185** by the loss of H_2_O, which further dissociated to produce a fragment ion at *m/z***129** and **86** due to elimination of (C_3_H_5_O_2_) ,(C_4_H_6_NO_3_), respectively. **B** = L-isoleucyl-L-proline[M + H]^**+**^with *m/z***228**), started the formation of a fragment ion at *m/z***210** by the loss of H_2_O, which further dissociated to produce a fragment ion at *m/z***86** due to the loss of the(C_6_H_8_NO_3_)

**Fig. 9:**
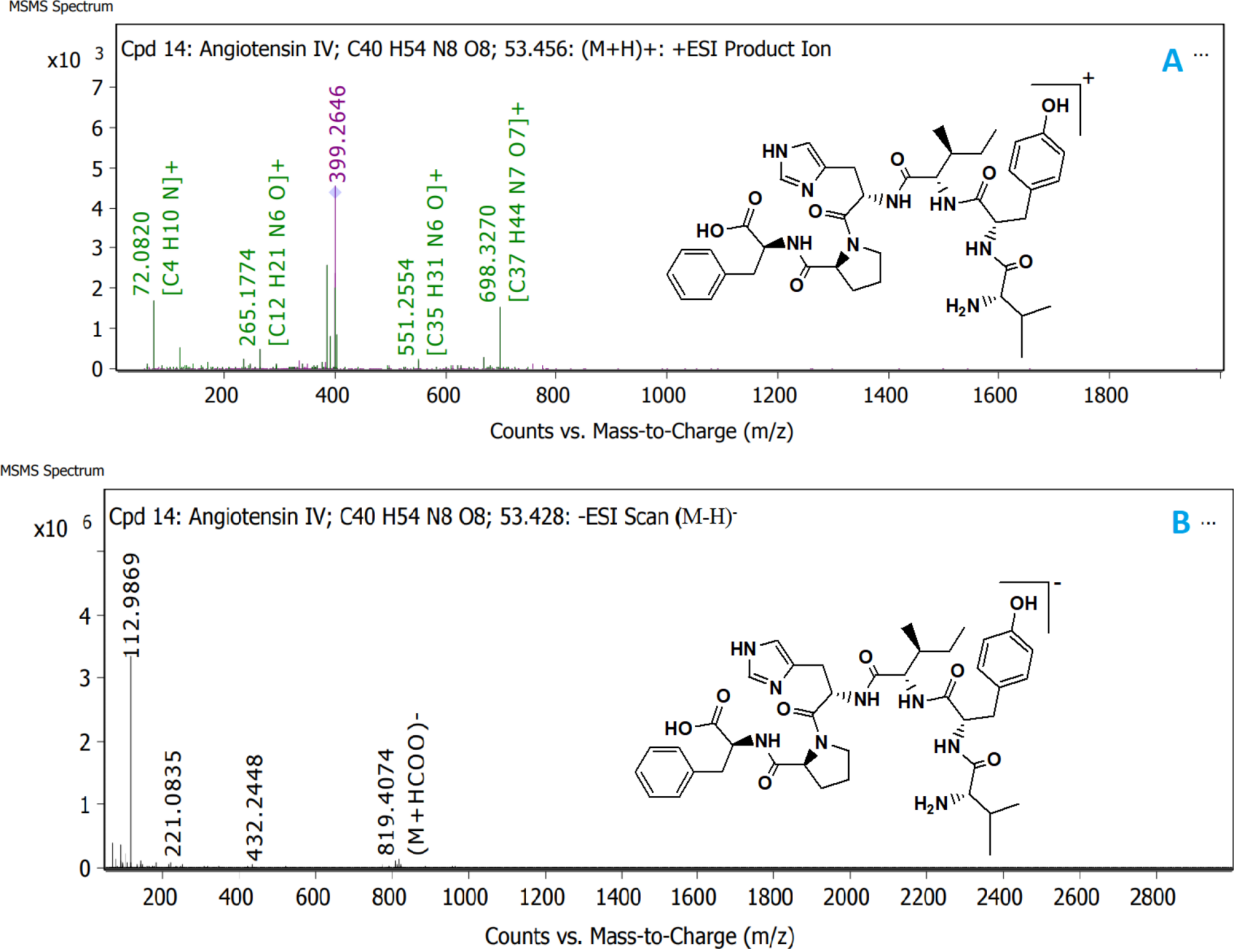
Proposed fragmentation pathways of main fragment ions for **Angiotensin IV** on the basis Q-TOF LC-MS/MS spectra in positive ionization mode [M + H]^**+**^ ions (**A**) and negative ionization mode [M − H]^**−**^ ions (**B**). See details produced **five** characteristic fragment ions at *m/z***698.3222**, at *m/z***551.2648**, at *m/z***399.2646** at *m/z***265.1774** and at *m/z***72.0826** for (**A**). Moreover, **three** characteristic fragment ions at *m/z***432.2448**, at *m/z***221.0835** and at *m/z***112.9869** were formed for (**B**)

Based on the results of medium B extract (Sh F) represented by its significant anti-inflammatory effects, prompted us to investigate its chemical composition with Q-TOF LC-HRMS spectroscopy analysis. In our study, the chemical composition of a highly bioactive extract (Sh F) from medium B was elucidated using Q-TOF LC-HRMS analysis. All compounds were identified depending on their m/z value from MS spectra in both positive and negative ionization modes ([M + H]^**+**^/[M-H]^**−**^, Fig. 7), using an Agilent LC/MS Mass Hunter Qualitative Software for preliminary identification, then confirmed from many other libraries databases (Figs. 8 and 9). In positive and negative ionization modes, Q-TOF LC-HRMS identified a total of 33 and 21 compounds, respectively (Table 2A, 2B).

### Q-TOF LC-HRMS analysis [positive ionization mode

According to our findings, the positive ionization mode was primarily characterized by the identified ions of peptides, fatty acids, amides, triglycerides, and others.

#### Peptides

The application of Q-TOF-MS/MS enabled the discovery of seven potentially bioactive peptides, including two isoleucyl-containing peptides, two phenylalanyl-containing peptides, two Histidinyl-Cysteine isomers, and the polypeptide “Angiotensin IV.” Their retention times (RT) were at 23.528, 27.265, 31.723, 33.901, 100.8, 101.92 and 53.456 min, respectively, and with molecular weights: 202.1323, 228.1483, 262.1327, 262.1329, 258.0784, 258.0785 and 774.4077 *m/z*, respectively. The Q-TOF-MS/MS product ions of these peptides are given in (Table 2A).

#### Structural fragmentation study of peptides

For isoleucyl-alanine and L-isoleucyl-L-proline; the complementary ions produced by the cleavage of the amino-alkyl group’s carbonyl C-C bond result in the characteristic fragment ion protonated “2-methylbutan-1-amine” with 86 *m/z* (C_5_H_12_N), Fig. 8).

Angiotensin IV has the molecular formula C_40_ H_54_ N_8_ O_8_. The positive and negative ion modes of its precursor [M + H]^**+**^ and [M-H]^−^ ions have *m/z* 775.4149 and 773.4022, respectively, with (RT) 53.456 and 53.428 min (Table 2A, 2B). The protonated and deprotonated angiotensin IV characteristic fragment ions were observed and identified in Fig. (9 A, 9B) (10 A, 10B).

The MS/MS spectrum in positive ionization mode shows three distinct pathways for fragmentation.

- **Pathway1**: producing the characteristic product ion at the *m/z* 399.2646 [M + H − C_20_H_30_N_3_O_4_]^+^.
- **Pathway 2**: showing fragments ions at *m/z* 265.1774 [M + H − C_26_H_35_N_6_O_5_]^+^, *m/z* 72.0826 [*m/z* 265.1774(C_14_H_19_N_2_O_3_) - C_10_H_10_NO_3_]^+^.
- **Pathway 3**: showing fragments ions at *m/z* 698.3222 [M + H − C_6_H_5_]^+^and at 551.2648 [698.3222(C_34_H_49_N_8_O_8_)- C_4_H9- C_6_H_5_O] ^+^.

**Fig. 10:**
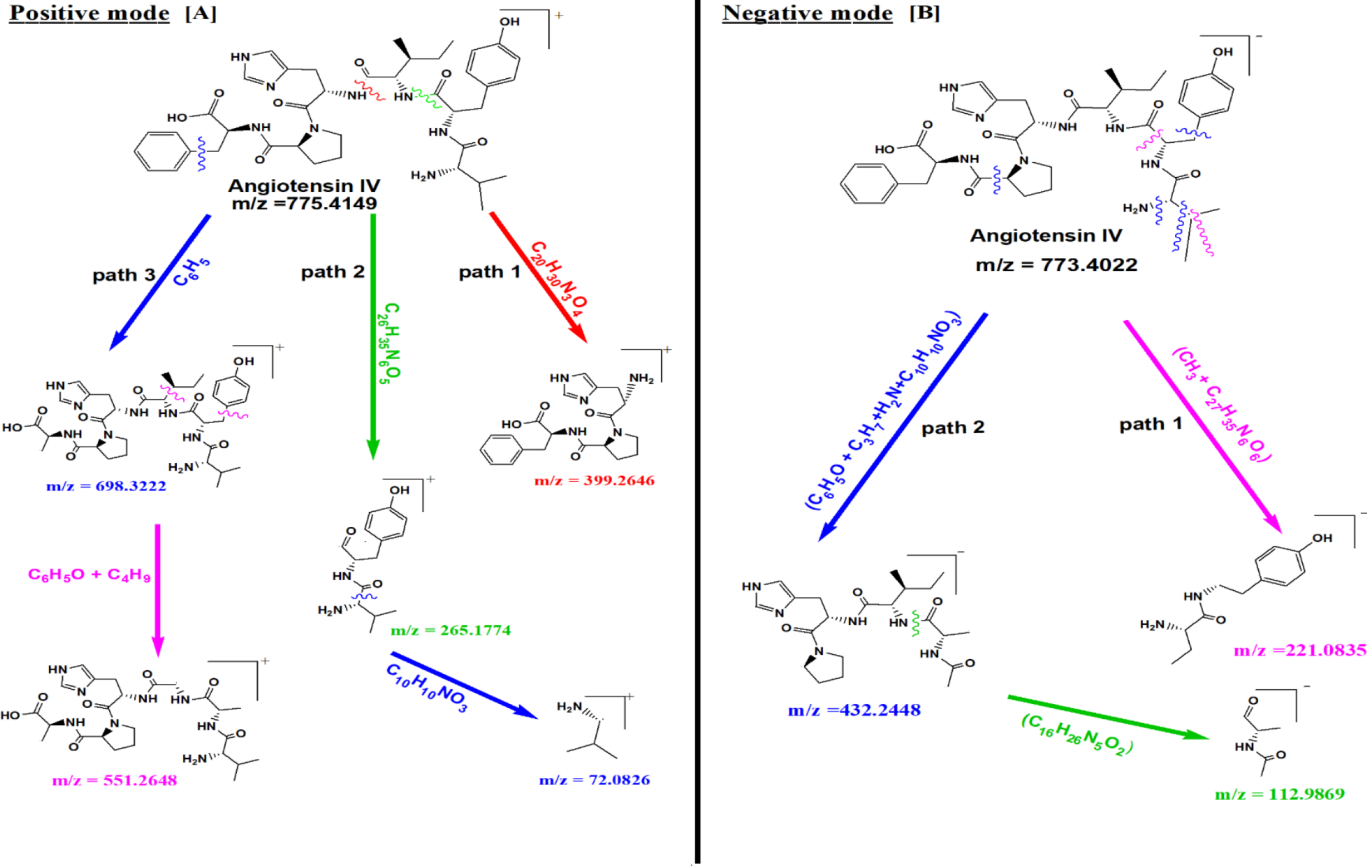
Comparison of the fragmentation pattern of the MS/MS spectrum demonstrated that **(A)** Protonated Angiotensin IV molecule give more informative and more intense peaks than do **(B)** deprotonated Angiotensin IV molecule

The data from the MS^3^ spectrum of Angiotensin IV could further confirm this conclusion (Figs. 10A and 9A, Table 2A). On the other hand, Angiotensin IV fragmentation in the negative ionization mode is followed by two pathways (Fig. 9B). Due to the loss of [M-H-C_27_H_35_N_6_O_6_-CH_3_]^**−**^, its characteristic minor fragment ion was observed at 221.0835 *m/z* in the first fragmentation pathway. Second fragmentation pathway: further fragmentation of precursor ion resulted in daughter ions at 432.2448 *m/z* due to loss of [M-H- C_6_H_5_O-C_3_H_7_-C_10_H_10_NO_3_-H_2_N]^−^ and 112.9869 *m/z* due the loss of [(C_16_H_26_N_5_O_2_)]^−^ from the fragment 432.2448[ (C_21_H_34_N_6_O_4_)]^−^ (Figs. 10B and 9B, Table 2B). The dissociation pattern of protonated Angiotensin IV [M + H]^+^molecule consists essentially of five peaks in positive mode and three peaks in deprotonated Angiotensin IV [M-H]^−^ molecule in Negative mode. (Figure 9A and B). Protonated Angiotensin IV molecules produce more informative and intense peaks than deprotonated Angiotensin IV molecules (Fig. 9A and B, Table 2A, 2B).

Bioactive peptides have been shown to have anti-inflammatory effect in macrophages by inhibiting the NO/iNOS and PGE2/COX-2 pathways while suppressing the production of pro-inflammatory cytokines such IL-1β and TNF-α [30]. When THP-1 cells were stimulated with TNF-α, Cysteine and Histidine reduced NF-κB activity [31]. It was confirmed that the polypeptide “Angiotensin IV” repressed inflammation in the brains of rats with chronic cerebral hypoperfusion (CCH), as it significantly reduced the levels of TNF-α, IL-1β, IL-6 and IL-12 in the brains of rats with CCH (CCH) [32].

#### Fatty acids

The use of Q-TOF-MS/MS allowed the identification of potential bioactive polyunsaturated fatty acids ; (2E,4E)-2,7-Dimethyl-2,4-octadienedioic acid, at *m/z* 198.0896 [M + H]^+^ (C_10_ H_14_ O_4_) (Rt 44.577 min), DB Diff. (-1.93); Methyl-18,18-dibromo-9E,17-octadecadien-5,7-diynoate, at *m/z* 442.0157 [M + H]^+^ (C_19_ H_24_ Br_2_ O_2_) (Rt 88.605 min) DB Diff. (-3.22); 8,12-Octadecadienoic acid at *m/z* 280.2422 [M + H]^+^ (C_18_ H_32_ O_2_) (Rt 89.069 min) DB Diff. (-6.97), in addition to Valproic acid at *m/z* 144.1161 [M + H]^+^ (C8 H16 O2) (Rt 94.533 min) DB Diff. (-1.75) (Table 2A).

In-vitro, saturated fatty acids (SFAs) were found to directly stimulate inflammatory gene expression via TLR4 signaling. The relative potency of various (SFAs) varied with chain length, with lauric acid (12:0) having the highest activity, whereas myristic acid (14:0) and stearic acid (18:0) having surprisingly little pro-inflammatory activity. Unlike SFAs, monounsaturated- fatty acids (MUFAs) and polyunsaturated fatty acids (PUFAs) did not activate TLR4 signaling. These researchers were able to demonstrate that pretreatment of cells for 3 h with a variety of PUFAs or oleic acid (18:1) significantly reduced the subsequent pro-inflammatory effect of lauric acid treatment. They went on to demonstrate that PUFAs’ ability to inhibit inflammatory responses induced by LPS or lauric acid was dependent on TLR4 [33]. Valproic acid (VPA) inhibits the CD45^high^F4/80^low^ macrophage subset as well as the production of pro-inflammatory cytokines/chemokines such as CXCL1, IL-5, IL-6, and IL-10. (VPA) specifically decreased tissue NF-кB2 p100 protein [34].

#### Amides

In Q-TOF LC-HRMS positive ionization mode analysis, eight amides were identified (Table 2A).

Anti-inflammatory activity has previously been reported for oleamide and anandamide (20:l, n-9).

The identified constituents were found at *m/z* 281.2736 [M + H]^+^ (C_18_ H_35_ NO) (Rt 94.533 min) DB Diff. (-1.75) and at *m/z* 353.3311[M + H]+ (C_22_ H_43_ NO_2_) (Rt 101.57 min) DB Diff. (-1.72), respectively.

**Fatty acid amides**, played critical roles in inflammation suppression [35]. Oleamide suppressed LPS-induced iNOS and COX-2 mRNA in RAW 264.7 cells, as well as inflammatory cytokines; TNF-α, IL-1β and IL-6 [36]. Anandamide inhibited NO and IL-6 release by LPS-stimulated J774 macrophages, as well as IL-12 and IL-23 production and increased IL-10 production by activated microglia via JNK and ERK1/2 activation and NF-κB [37].

#### Other compounds

In addition; ten other compounds were identified, three of which were previously reported to have anti-inflammatory effect; **Hydroxy-ibuprofen**, at *m/z* 222.1267 [M + H]^+^ (C_13_ H_18_ O_3_) (Rt 62.635 min) DB Diff. (-1.07), **Resveratrol** at *m/z* 228.0797 [M + H]^+^ (C_14_ H_12_ O_3_) (Rt 68.579 min) DB Diff. (-1.06) and **Rifamycin-O** at *m/z* 753.2991 [M + H]^+^ (C_39_ H_47_ N O_14_) (Rt 125.81 min) DB Diff. (0.59) (Table 2A).

**Hydroxy Ibuprofen** is an Ibuprofen metabolite. Ibuprofen is an anti-inflammatory agent with IC50s of 13 M and 370 M for COX-1 and COX-2, respectively [38]. **Resveratrol** was a potent inhibitor of the NO and cytokine release in activated macrophages and microglia [39]. **Rifamycin-O** also antagonized TNF-α and LPS-induced NF-κB activities and inhibited IL1β-induced synthesis of inflammatory chemokine, IL8 [40].

### Q-TOF LC-HRMS analysis [negative ionization mode

Furthermore, negative ionization mode was mainly characterized by the identification of fatty acids, phenolic and glycoside compounds (Table 2B).

#### Peptides

Two peptides were only identified in negative ionization mode: Angiotensin IV and Se-Adenosylseleno-homocysteine. In addition, the amino acid; **5-methoxy-DL-Tryptophan** with Rt (33.421 min), M Wt. (*m/z* 234.1019, C_12_ H_14_ N_2_ O_3_), DB Diff. (-6.04) was identified (Table 2B). 5-methoxy-DL-Tryptophan reduces LPS-induced expression of COX-2, TNF-α, IL-1β, and IL-6 in RAW 264.7 macrophages [41].

#### Fatty acids

Five fatty acids were identified, one of which was Isopropyl-3-(3,4-dihydroxyphenyl)-2-hydroxypropanoate (**IDHP**); with Rt (43.097 min), M Wt. (*m/z* 240.1013, C_12_ H_16_ O_5_), DB Diff. (-6.51) (Table 2B). **(IDHP)** drastically reduced NO, TNF-α, and IL-1β production in LPS-induced BV-2 cells and rat primary microglia. IDHP also suppressed LPS-induced iNOS, TNF-α, and IL-1β mRNA expression [42].

#### Halogenated, phenolic compounds and glycosides

Furthermore, eleven compounds were identified in negative ionization mode, from which the followings : **Granisetron**, Rt (63.003 min), M Wt.( *m/z* 312.1955, C_18_ H_24_ N_4_ O), DB Diff. (-1.69), **Fenofibric acid**, Rt (68.719 min), MWt.(*m/z* 318.0679, C_17_H_15_ClO_4_), DB Diff. (-6.4), **Umbelliprenin**, Rt (72.239 min), M Wt.( *m/z* 366.2186, C_24_ H_30_ O_3_), DB Diff. (2.49**)**,** Alpha-CEHC**, Rt ( 74.282 min), M Wt.( *m/z* 278.1537, C_16_ H_22_ O_4_), DB Diff. (-6.31) and **Piceid**, Rt (66.684 min), M Wt.( *m/z* 390.1336, C_20_ H_22_ O_8_), DB Diff. (-5.53) (Table 2B), were previously reported to have anti-inflammatory activity.

The amide **“Granisetron”** significantly reduced TNF-a, IL-6, HMGB1, and NF-kB. It also reduced the expression of the receptor for advanced glycation end, TLR4, in liver tissue, as well as pyroptosis, by decreasing NLRP3, IL-1β, and caspase-1 [43]. **Fenofibrate** decreases the expression and secretion of TNF-α, IL-1β, and IL-6 via the NF-κB signaling pathway, making them therapeutic targets for attenuating inflammation in hepatic pathological progression [44].

**Umbelliprenin** (UMB), a natural sesquiterpene coumarin, may reduce inflammation by lowering IL-17 levels in the blood. **UMB** exerts its anti-inflammatory effects by modulating distinct cytokine release/inhibition types [45]. **Alpha-CEHC** is a vitamin E derivative that reduces LPS-induced gene expression of TNF-α, IL-1β, IL-6, and iNOS. While high concentrations increased gene expression in peritoneal macrophages [46]. Studies in rat models have indicated that vitamin E exhibits anti-inflammatory properties by suppressing IL-1 and IL-6. Vitamin E has also been shown to inhibit COX-2, the enzyme involved in inflammatory reactions [47]. **Piceid** is a Resveratrol glycoside: Resveratrol inhibited NO and cytokine release in activated macrophages and microglia [39].

**Table 2:** Characterization of secondary metabolites from “*A. Unguis* isolate SP51-EGY” fungal extract by Q-TOF LC-HRMS analysis (A = positive, B = negative ionization mode)

	Proposed Compounds [A]	RT (min)	Molecular Formula	Molecular Weight (m/z)	Fragment ions	Mass Error (ppm)	Ionization ESI (+)	Reference
	**Peptides**							
1	Isoleucyl-Alanine	23.528	C_9_ H_18_ N_2_ O_3_	202.1323	185.1284, 156.9625, 86.0971	-0.57	[M-H_2_O + H]^+^	[48]
2	L-isoleucyl-L-proline	27.265	C_11_ H_20_ N_2_ O_3_	228.1483	211.1450, 183.0928	-0.9	[M-H_2_O + H]^+^	HMDB0011174
3	L-phenylalanyl-L-proline	31.723	C_14_ H_18_ N_2_ O_3_	262.1327	245.1293, 217.1071	-0.92	[M-H_2_O + H]^+^	HMDB0011177
4	L-prolyl-L-phenylalanine	33.901	C_14_ H_18_ N_2_ O_3_	262.1329	245.1295, 217.1013, 70.0663, 60.0840	-1.12	[M-H_2_O + H]^+^	HMDB0011179
5	**Angiotensin IV** [H-Val-Tyr-Ile-His-Pro-Phe-OH]	53.456	C_40_ H_54_ N_8_ O_8_	774.4077	775.4149, 676.3474, 497.2835, 399.2646, 265.1774, 236.1617, 72.0820	-1.58	[M + H]^+^	[48]
6	Histidinyl-Cysteine	100.8	C_9_ H_14_ N_4_ O_3_ S	258.0784	241.0754, 223.0647	0.99	[M-H_2_O + H]^+^	[48]
7	Cysteinyl-Histidine	101.92	C_9_ H_14_ N_4_ O_3_ S	258.0785	241.0754,223.0647	0.47	[M-H_2_O + H]^+^	HMDB0028777 [48]
	**Fatty acids**							
8	(2E,4E)-2,7-Dimethyl-2,4-octadienedioic acid	44.577	C_10_ H_14_ O_4_	198.0896	181.0863, 156.1209, 129.0530, 115.9653, 95.9737,	-1.93,	[M-H_2_O + H]^+^	[48]
9	Methyl 18,18-dibromo-9E,17 -octadecadien-5,7-diynoate	88.605	C_19_ H_24_ Br_2_ O_2_	442.0157	443.0238, 425.2169, 352.3414,	-3.22	[M + H]^+^	(CFM-ID)
10	*8*,*12-octadecadienoic acid*	89.069	C_18_ H_32_ O_2_	280.2422	280.2654, 263.2383, 95.0864	-6.97	[M-H_2_O + NH_4_]^+^	[49]
11	Butyl butyrate	93.044	C_8_ H_16_ O_2_	144.1161	145.1229, 127.1128	-7.1	[M-H_2_O + H]^+^	[48]
12	Valproic acid	94.929	C_8_ H_16_ O_2_	144.1161	127.1129,	-1.1	[M-H_2_O + H]^+^	[50]
	**Amides**							
13	Palmitic amide	92.832	C_16_ H_33_ N O	255.2579	256.2615, 124.0879	-6.11	[M + H]^+^	[48]
14	Oleamide	94.533	C_18_ H_35_ NO	281.2736	282.2808, 127.1128	-1.75	[M + H]^+^	[49]
15	N-oleoyl alanine	95.485	C_21_ H_39_ N O_3_	353.2946	352.3416, 336.2913, 282.2807, 127.1128	-4.53	[M-H_2_O + H]^+^	[49]
16	Sphingosine	95.547	C_18_ H_37_ N O_2_	299.2837	282.2807, 124.0879	-4.34	[M-H_2_O + H]^+^	MassBank of Japan (https://www.mssj.jp/), HMDB0000252
17	D-erythro-Sphingosine C-20	100.6	C_20_ H_41_ N O_2_	327.3154	352.3413, 310.3121, 309.3143, 284.2964,	-5.16	[M-H_2_O + H]^+^	(CFM-ID)
18	Stearamide	100.9	C_18_ H_37_ N O	283.2891	284.2965, 223.0648, 124.0877, 116.1076	-5.73	[M + H]^+^	[48]
19	Anandamide (20:l, n-9)	101.57	C_22_ H_43_ NO_2_	353.3311	336.3268, 317.3032, 223.0646,100.0765	-4.87	[M-H_2_O + H]^+^	HMDB0031678 [48]
20	Stearoylethanolamide	101.61	C_20_ H_41_ N O_2_	327.315	310.3121, 284.2964, 124.0878	-3.85	[M-H_2_O + H]^+^	[48]
	**Triglycerides**							
21	MG(16:0/0:0/0:0)	95.338	C_19_ H_38_ O_4_	330.2785	353.2683, 331.2892, 313.2755	-4.49	[2 M + Na]^+^	HMDB0011533 (CFM-ID)
22	2-(14,15-Epoxyeicosat-rienoyl) Glycerol	102.5	C_23_ H_38_ O_5_	394.2735	352.3412, 285.0956, 223.0674	-3.87	[M-H_2_O + H]^+^	[48]
23	Triricinolein (triglyceride)	137.45	C_57_ H_104_ O_9_	932.7694	951.7786, 933.7768, 657.5075, 519.1396, 321.2411, 285.0956	-1.44	[M + NH_4_]^+^	[48]
	**Others**							
24	Lyciumoside IV [acyclic diterpene glycoside]	55.225	C_38_ H_64_ O_16_	776.4243	759.4209, 380.2147, 315.1830	-6.29	[M-H_2_O + H]^+^	HMDB0033499 [48]
25	Hydroxy ibuprofen	62.635	C_13_ H_18_ O_3_	222.1267	227.1053, 205.1232, 187.1127, 157.0480	-4.8	[M-H_2_O + Na]^+^	[48]
26	trans-Resveratrol	68.579	C_14_ H_12_ O_3_	228.0797	233.0583, 211.0765, 197.9894, 185.0427	-4.63	[M-H_2_O + Na]^+^	[48]
27	Eszopiclone	80.271	C_17_ H_17_ Cl N_6_ O_3_	388.1059	411.0957, 389.1132, 371.1047, 352.3411, 129.0533	-2.27	[M + Na]^+^	[48]
28	Zopiclone	82.176	C_17_ H_17_ Cl N_6_ O_3_	388.1058	411.0954, 389.1133, 371.1094, 352.3410, 129.0532	-1.8	[M + Na]^+^	[48]
29	Methenolone	89.075	C_20_ H_30_ O_2_	302.2237	302.2472, 167.0564	0.87	[M-H_2_O + NH_4_]^+^	HMDB0041928 [48]
30	2,4,6-Triethyl-1,3,5-trithiane	101.92	C_9_ H_18_ S_3_	222.0574	223.0647, 124.0877	-0.36	[M + H]^+^	[48]
31	Rifamycin O	125.81	C_39_ H_47_ N O_14_	753.2991	772.2763, 667.1737, 519.1405, 223.0647	0.78	[M-H_2_O + K]^+^	(CFM-ID)
32	Uroporphyrin I	139.85	C_40_ H_38_ N_4_ O_16_	830.2347	848.2731, 832.2411, 758,2226	-7.73	[M + NH_4_]^+^	[48]
33	Vitisifuran A	150.31	C_56_ H_40_ O_12_	904.252	124.0877	0.02	[M + NH_4_]^+^	HMDB0034785 [48]
	**Proposed Compounds** **[B]**	**RT** **(min)**	**Molecular Formula**	**Molecular** **Weight** **(** *m/z* **)**	**Fragment ions**	**Mass Error** **(ppm)**	**Ionization** **ESI (-)**	**Reference**
	**Peptides**							
1	5-Methoxy-DL-tryptophan	33.421	C_12_ H_14_ N_2_ O_3_	234.1019	215.0841, 112.9868	-6.04	[M-H_2_O-H]^−^	[48]
2	Angiotensin IV	53.428	C_40_ H_54_ N_8_ O_8_	774.4093	433.256, 221.129, 114.056	-3.67	[M + HCOO]^−^	Manually confirmed
3	Se- Adenosylseleno-homo- cysteine	84.53	C_14_ H_20_ N_6_ O_5_ Se	426.0666	407.0482 ,248.9631	5.41	[M-H_2_O-H]^−^	HMDB0011117 [48]
	**Fatty acids**							
4	Isopropyl 3-(3,4-dihydroxy phenyl)-2-hydroxypropanoate	43.097	C_12_ H_16_ O_5_	240.1013	221.0835, 112.9868	-6.51	[M-H_2_O-H]^−^	[48]
5	3-carboxy-4-methyl-5-propyl-2-furanpropanoic acid	44.172	C_12_ H_16_ O_5_	240.1014	238.9334, 221.0836	-6.86	[M-H_2_O-H]^−^	HMDB0061112 [48]
6	(2E,4E)-2,7-Dimethyl-2,4-octadienedioic acid	44.529	C_10_ H_14_ O_4_	198.0907	198.0907,179.0728	-7.66	[M-H_2_O-H]^−^	HMDB0034099 [48]
7	18,18-dibromo-9E,17-octadecadien-5,7-diynoic acid	77.759	C_18_ H_24_ Br_2_ O_2_	428	426.9936, 248.9628	-3.18	[M-H]^−^	(CFM-ID)
8	9R,10 S-dihydroxy-stearic acid	95.305	C18 H36 O4	316.2636	316.2636, 112.9869	-2.2	[M + CH3COO]-	HMDB0302281 [49]
	**Triglyceride**							
9	MG(0:0/18:0/0:0) [monoglyceride]	101.66	C_21_ H_42_ O_4_	358.3105	403.3084,358.3105	-5.98	[M + HCOO]^−^	[48] HMDB0011535
10	Triricinolein (triglyceride)	137.32	C_57_ H_104_ O_9_	932.772	,297.2455,	-4.24	[M + HCOO]^−^	HMDB0038061 [48]
	**Halogenated**,** phenolic compounds and glycosides**							
11	5-Hydroxythalidomide	60.829	C_13_ H_10_ N_2_ O_5_	274.0607	255.0447,	-1.71	[M-H_2_O-H]^−^	HMDB0013871 [48]
12	Granisetron	63.003	C_18_ H_24_ N_4_ O	312.1955	293.1776	-1.69	[M-H_2_O-H]^−^	[48]
13	Sulfasalazine	67.065	C_18_ H_14_ N_4_ O_5_ S	398.0692	398.0692,371.1158	-0.71	[M + HCOO -H_2_O]^−^	HMDB0014933 [49]
14	Fenofibric acid	68.719	C_17_ H_15_ Cl O_4_	318.0679	359.0714,	-6.4	[M + CH_3_COO - H_2_O]^−^	HMDB0252207 MassBank of Japan (https://www.mssj.jp/)
15	Umbelliprenin [coumarin]	72.239	C_24_ H_30_ O_3_	366.2186	383.1782, 347.2017, 248.9632, 112.9868	2.49	[M + Cl -H_2_O]^−^	[48]
16	Alpha-CEHC (alpha-Carboxyethylhydroxy-chroman)	74.282	C_16_ H_22_ O_4_	278.1537	277.1464, 248.6929	-6.31	[M-H]^−^	[48]
17	4-Dodecylbenzenesulfonic acid	82.422	C_18_ H_30_ O_3_ S	326.1936	325.1863, 293.1811,	-6.24	[M-H]^−^	[49]
18	Nafcillin	82.982	C_21_ H_22_ N_2_ O_5_ S	414.1253	459.1240, 415.0301, 325.1864,	-0.36	[M + HCOO]^−^	[48]
19	Persicachrome	95.311	C_25_ H_36_ O_3_	384.2641	384.2641,365.2487	2.31	[M-H_2_O-H]^−^	HMDB0036425 [48]
20	Lyciumoside IV, [acyclic diterpene glycoside]	55.207	C_38_ H_64_ O_16_	776.4251	803.4128,776.4251	-7.3	[M + HCOO-H_2_O]^−^	HMDB0033499 [49]
21	Piceid (Resveratrol glycoside)	66.684	C_20_ H_22_ O_8_	390.1336	390.1336, 371.1158	-5.53	[M-H_2_O-H]^−^	HMDB0031422 [48]

## Conclusions

The intricate interplay of TLR4 in immune responses underscores its dual role as both protector and potential provocateur. Malfunctioning TLR4 signaling can inadvertently amplify immune responses, thereby inducing conditions like sepsis, acute lung injury, and pathological chronic inflammation, often linked to cancer and autoimmune maladies. Central to this inflammatory response is the production of NO by iNOS, which triggers the subsequent activation TNF-α, and IL-6. Notably, this study delved into the potential Nrf2-dependency of the anti-inflammatory properties of “*Aspergillus unguis* isolate SP51-EGY” extracts, revealing their effects to be independent of Nrf2 modulation. A significant modulation in the expression of inflammatory markers, such as iNOS, COX-2, TNF-α, and IL-6 was observed using real-time qPCR. The extract labeled (B Sh F) was particularly potent in this regard. Further chemical profiling of this extract via Q-TOF LC-HRMS unveiled a rich tapestry of 54 bioactive compounds, with several playing pivotal roles in inflammation suppression. Key compounds such as granisetron, fenofibrate, and umbelliprenin were shown to attenuate inflammatory pathways, particularly the NF-κB signaling cascade. Marine-associated fungi, like the “*Aspergillus unguis* isolate SP51-EGY” from the Red Sea, have emerged as bounteous reservoirs of secondary metabolites, displaying diverse biological activities and intricate structures. This study not only underscores the potential of LC-HRMS as a formidable analytical tool but also highlights the pressing need for further research into the bioaccessibility, bioavailability, and toxicological profiles of these fungal extracts. Future endeavors should also encompass animal model studies, paving the way for the potential therapeutic commercialization of these promising extracts.

## Electronic supplementary material

Below is the link to the electronic supplementary material.

Supplementary Material 1

## Data Availability

The raw data supporting the conclusions of this article will be made available by the authors, without unjustified reservation.

## Abbreviations

COX-2: Cyclooxygenase-2
ELISA: Enzyme-linked immunosorbent assay
HO-1: Heme oxygenase 1
iNOS: Inducible nitric oxide synthase
IL-1: Interleukin-1
IL-6: Interleukin-6
LPS: Lipopolysaccharide
LC-HRMS: Liquid chromatography-high-resolution mass spectrometry
NO: Nitric oxide
Nrf2: Nuclear factor erythroid 2–related factor 2
NF-κB: Nuclear factor-kappa B
OSGIN1: Oxidative stress induced growth inhibitor 1
PUFAs: Polyunsaturated fatty acids
qPCR: Quantitative real-time polymerase chain reaction
TLRs: Toll-Like Receptors
TNF-α: Tumor necrosis factor alpha
UMB: Umbelliprenin
