# Supplementary material for: Anti-inflammatory potential of aspergillus unguis SP51-EGY: TLR4-dependent effects & chemical diversity via Q-TOF LC-HRMS
Source: BMC Biotechnol. 2024 Sep 18;24:62. doi: 10.1186/s12896-024-00890-1 (PMC11411751; doi:10.1186/s12896-024-00890-1)
Supplement: Supplementary file 1 — Supplementary Material 1 [file 12896_2024_890_MOESM1_ESM.docx]

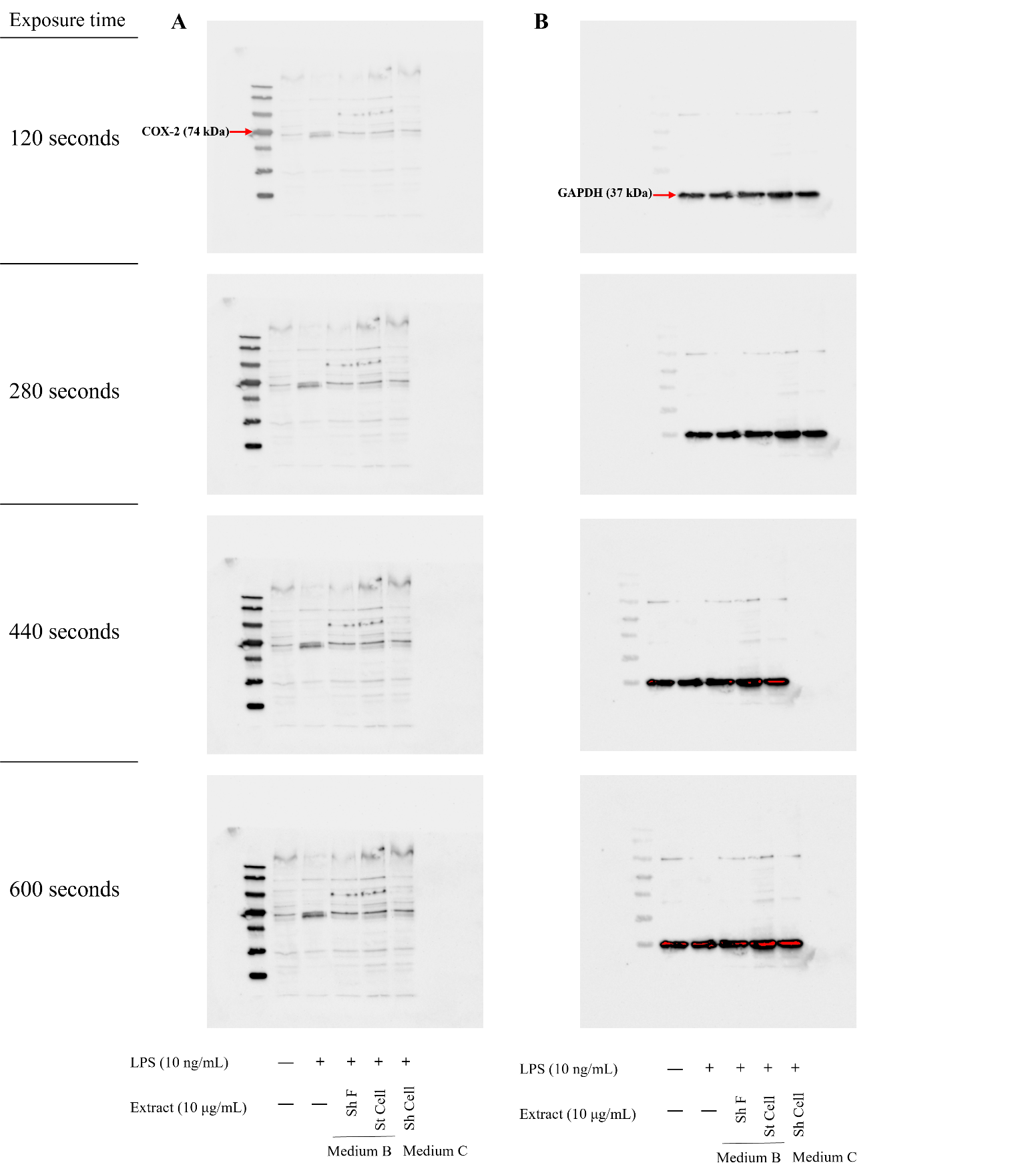


**Supplementary Figure 1. Full uncropped Western blots corresponding to the data presented in Figure 6.** This figure displays Western blot data comparing protein expression levels of COX-2 (A), and GAPDH (B) as a loading control across different samples. Each lane represents a distinct sample and loading controls (GAPDH) were run on the same blot for accurate normalization. Multiple exposures were captured at 120, 280, 440, and 600 seconds to ensure optimal signal visualization without overexposure. Quantitative comparisons between samples were exclusively made using data derived from the same blot.
